# Herpud1 negatively regulates pathological cardiac hypertrophy by inducing IP3 receptor degradation

**DOI:** 10.1038/s41598-017-13797-z

**Published:** 2017-10-17

**Authors:** Natalia Torrealba, Mario Navarro-Marquez, Valeria Garrido, Zully Pedrozo, Diego Romero, Yuka Eura, Elisa Villalobos, Juan Carlos Roa, Mario Chiong, Koichi Kokame, Sergio Lavandero

**Affiliations:** 1 Advanced Center for Chronic Disease (ACCDiS) & Center for Molecular Studies of the Cell (CEMC), Facultad de Ciencias Químicas y Farmacéuticas & Facultad de Medicina, Santiago, Chile; 2 Instituto de Ciencias Biomédicas, Facultad de Medicina Universidad de Chile, Santiago, Chile; 3 Department of Pathology, Advanced Center for Chronic Diseases (ACCDiS), Faculty of Medicine, Pontifical Catholic University of Chile, Santiago, Chile; 4 Department of Molecular Pathogenesis, National Cerebral and Cardiovascular Center, Suita, Osaka, Japan; 5 Department of Internal Medicine (Cardiology Division), University of Texas Southwestern Medical Center, Dallas, Texas USA

## Abstract

Cardiac hypertrophy is an adaptive response triggered by pathological stimuli. Regulation of the synthesis and the degradation of the Ca^2+^ channel inositol 1,4,5-trisphosphate receptor (IP3R) affects progression to cardiac hypertrophy. Herpud1, a component of the endoplasmic reticulum-associated degradation (ERAD) complex, participates in IP3R1 degradation and Ca^2+^ signaling, but the cardiac function of Herpud1 remains unknown. We hypothesize that Herpud1 acts as a negative regulator of cardiac hypertrophy by regulating IP3R protein levels. Our results show that Herpud1-knockout mice exhibit cardiac hypertrophy and dysfunction and that decreased Herpud1 protein levels lead to elevated levels of hypertrophic markers in cultured rat cardiomyocytes. In addition, IP3R levels were elevated both in Herpud1-knockout mice and Herpud1 siRNA-treated rat cardiomyocytes. The latter treatment also led to elevated cytosolic and nuclear Ca^2+^ levels. In summary, the absence of Herpud1 generates a pathological hypertrophic phenotype by regulating IP3R protein levels. Herpud1 is a novel negative regulator of pathological cardiac hypertrophy.

## Introduction

Pathological cardiac hypertrophy is a phenotypic alteration of the heart to compensate for loss of function after myocardial infarction or in association with chronic stress, as in hypertension^1–3^. Cardiomyocytes are terminally-differentiated cells that are responsible for myocardial contraction. In response to pro-hypertrophic stimuli, cardiomyocytes activate signaling pathways such as calcineurin/NFAT that favor their growth and increase their contractile function^4^. Activation of these pathways triggers reprogramming of gene expression in cardiomyocytes, including fetal genes such as the beta-myosin heavy chain (*Myhβ*)^5^. These processes initially serve to enhance cardiomyocyte performance but eventually lead to contractile dysfunction and cell death by apoptosis^6^. At the tissue level, these events may also lead to interstitial fibrosis^7^ and decreased vascular density^8^. Therefore, although hypertrophy is initially an adaptive response to increased demand, sustained hypertrophy may progress into heart failure^9^. Developing therapies that might prevent or reverse pathological hypertrophy, thereby avoiding progression to heart failure, requires an improved understanding of the mechanisms that lead to cardiomyocyte hypertrophy and identification of novel therapeutic targets.

Intracellular Ca^2+^-signaling pathways play a key role in the cardiac hypertrophy^10^, along with proteins that regulate Ca^2+^, including the inositol trisphosphate receptor (IP3R)^11,12^. IP3R is a tetrameric Ca^2+^ channel found in the endoplasmic reticulum (ER) membrane and nuclear envelope^13,14^. Three isoforms of IP3R have been described, IP3R1, IP3R2, and IP3R3, each of which forms homo- or hetero-tetramers with different properties and tissue distributions. IP3R2 is the predominant isoform in the heart^15^. Overexpression of IP3R2 induces cardiomyocyte hypertrophy, favoring a release of Ca^2+^ from the ER^11,12^ that is independent of the Ca^2+^ release required for contraction. This Ca^2+^ release activates pro-hypertrophic signaling pathways such as calcineurin/NFAT^4,11,16^. Thus, regulation of both the synthesis and the degradation of IP3R may be relevant to the genesis and progression of cardiac hypertrophy.

Herpud1 is an ER membrane protein, originally described as a target in the unfolded protein response (UPR), as its levels rise significantly in response to various stressors^17^. Herpud1 was subsequently reported to facilitate the retro-translocation of proteins from the ER to the 26 S proteasome in the cytosol for proteolytic elimination^18–20^. IP3R1 has been shown to be a Herpud1 target^20^ with the ability to regulate intracellular Ca^2+^ levels in neuron and HeLa cell lines^20,21^. This ability suggests a potential role in cardiac IP3R degradation, which occurs via the proteasome pathway^22^. Although the presence of Herpud1 mRNA has been described in cardiac tissue^17^, the expression and physiological function of the Herpud1 protein in the heart are unclear, and its potential role in cardiac hypertrophy remains unexplored.

Given the above, we hypothesized that Herpud1 regulates IP3R degradation in the cardiomyocyte, acting as a negative regulator of cardiac hypertrophy. The goal of this study was to elucidate the role of Herpud1 in cardiac pathophysiology and to assess whether Herpud1 might serve as a novel therapeutic target in pathological cardiac hypertrophy.

## Results

### Herpud1 is present in the mouse heart

**Figure 1:**
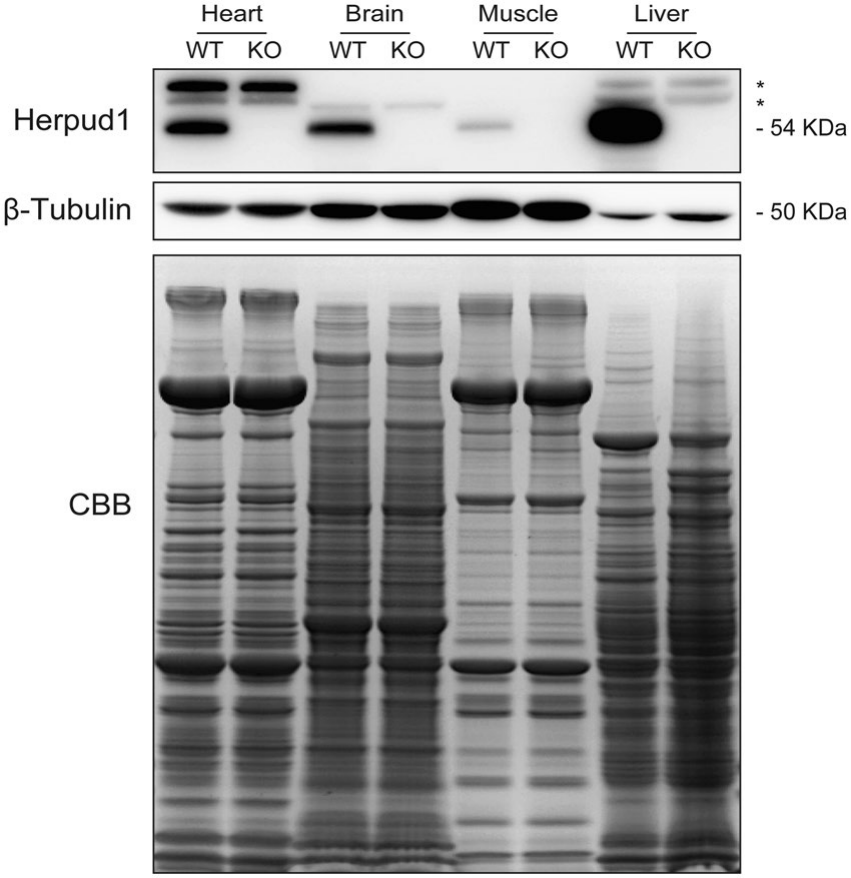
Expression of Herpud1 protein in mouse tissues. Representative Western blot of Herpud1 protein levels in heart, brain, skeletal muscle (*extensor digitorum longus*), and liver samples from wild-type (WT) and Herpud1-knockout (KO) mice (n = 3). *Non-specific band.

Cardiac hypertrophy is a pathological process favored by changes in Ca^2+^ signaling. Cytoprotective proteins such as Herpud1 may regulate these signals, mainly by inducing degradation of ER proteins and maintaining Ca^2+^ homeostasis^20,21^. To date, the presence of Herpud1 mRNA has only been reported in human cardiac tissue^17^. To elucidate whether the Herpud1 protein is present in mouse cardiac tissue, we studied 10-to-12-week-old C57BL/6 wild-type (WT) and Herpud1-knockout (KO)^23^ male mice (Supplementary Fig. 1). Samples were obtained from the heart, brain, liver, and skeletal muscle of the mice. The Herpud1 protein was found in these tissues in WT mice but was completely absent in the tissues from Herpud1-KO mice (Fig. 1). The distribution of Herpud1 in the adult heart in WT and KO mice as well as in NRVM was studied by immunohistochemistry and immunocytochemistry (Supplementary Fig. 2), These results provide the first evidence of the presence and distribution of Herpud1 in the mouse heart. The relative expression of Herpud1 mRNA was also assessed in the cardiac tissue samples and was found to be expressed in WT animals but not Herpud1-KO mice (Supplementary Table 1).

### Herpud1-KO mice develop cardiac hypertrophy and systolic dysfunction

**Figure 2:**
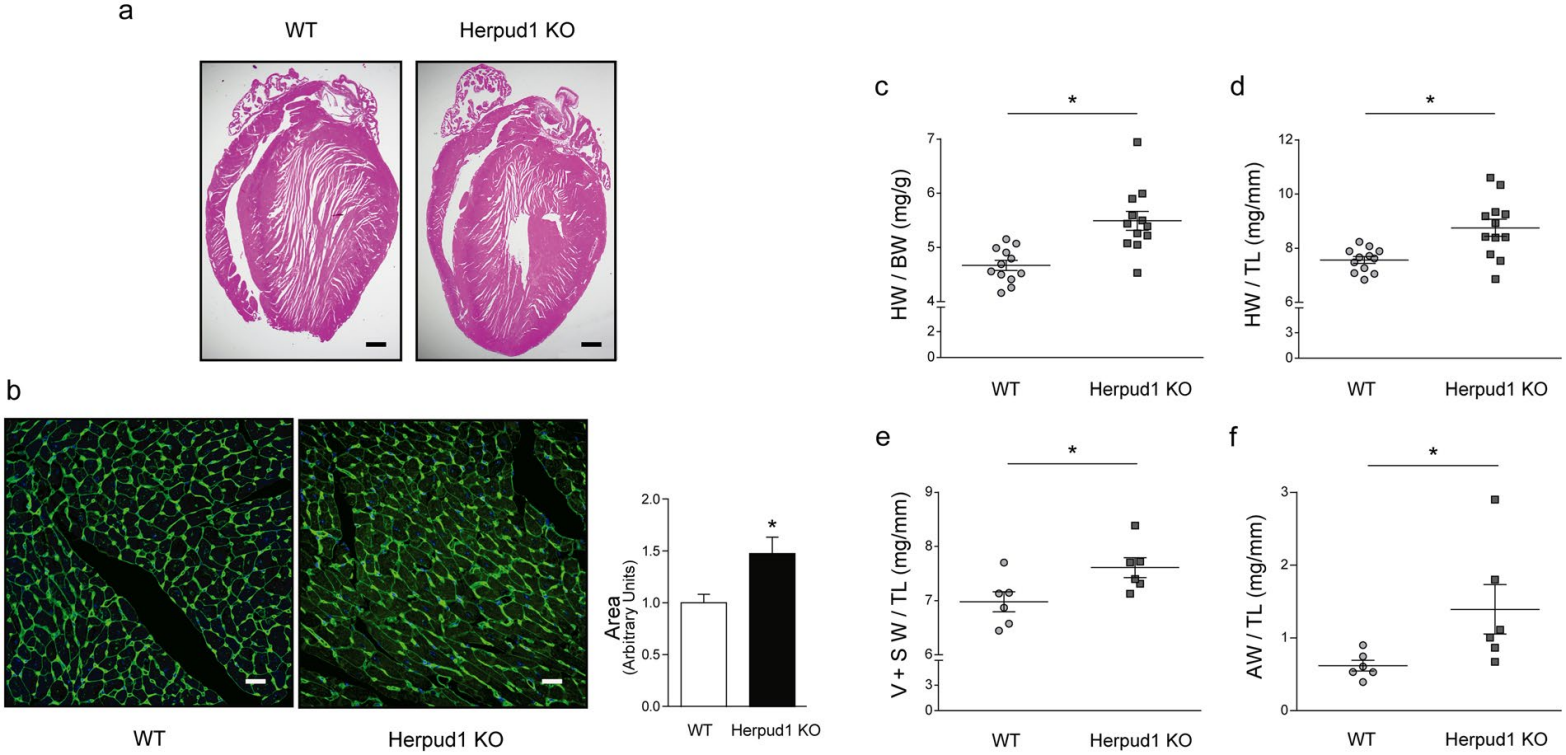
Herpud1-KO mice develop cardiac hypertrophy. (**a**) Four-chamber image, 40X, with hematoxylin and eosin staining (n = 3). (**b**) Representative images (400X) of paraffin embedded cardiac tissue stained with wheat germ agglutinin (WGA) and quantification of cardiomyocyte cross-sectional area (n = 6). (**c**) Heart weight/body weight (mg g^−1^, n = 12). (**d**) Heart weight/tibia length (mg cm^−1^, n = 12). (**e**) Left and right ventricular plus septum weight/tibia length (mg cm^−1^, n = 6). (**f**) Left and right atrial weight/tibia length^−1^ (mg cm^−1^, n = 6) in wild-type (WT) and Herpud1-knockout (Herpud1-KO) 10-to-12-week-old mice. Mean ± SEM, analyzed using a *t*-test. *p < 0.05 vs. WT.

**Figure 3:**
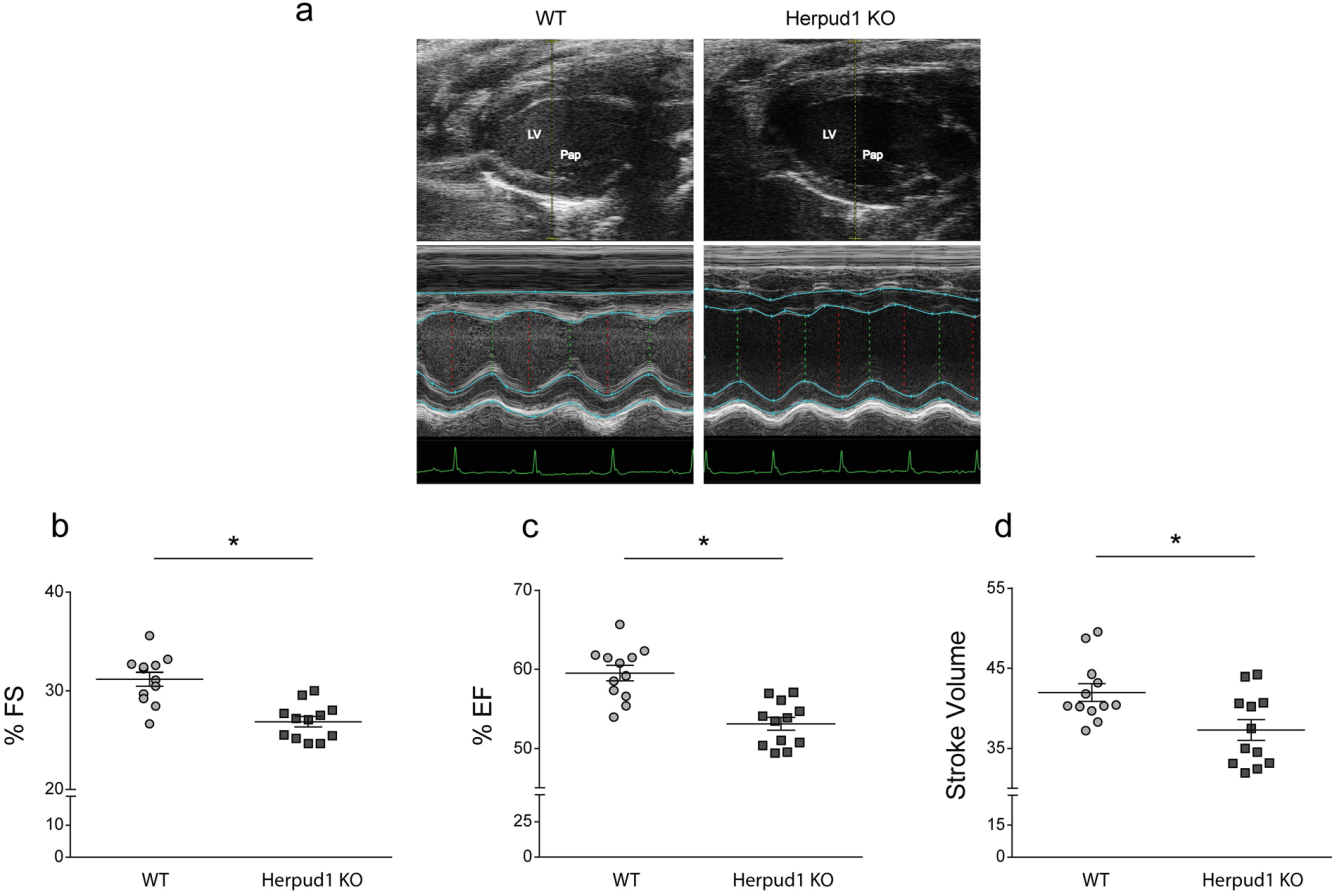
Herpud1-KO mice develop myocardial dysfunction. (**a**) Representative long-axis echocardiography images, 2D and M-mode, of the left ventricle. LV: Left ventricle, Pap: papillary muscle. (**b**) % Fractional shortening (n = 12). (**c**) % Ejection fraction (n = 12). (**d**) Stroke volume (n = 12) in wild-type (WT) and Herpud1-knockout (Herpud1 KO) 10-to-12-week-old mice. Mean ± SEM, analyzed using a *t*-test. *p < 0.05 vs. WT.

Morphological studies showed significantly elevated heart mass in Herpud1-KO mice as compared to their WT counterparts, as measured by heart weight/total body weight (Fig. 2a–c) and heart weight/tibia length (Fig. 2d) ratios. Similarly, the left ventricular + septum weight/tibia length (Fig. 2e) and atrial weight/tibia length (Fig. 2f) ratios were elevated in Herpud1-KO group as compared to WT group. In agreement with the development of cardiac hypertrophy, cardiomyocyte cross-sectional area was significantly increased in Herpud1 silenced mice (Fig. 2b). We also assessed the relative levels of genes expressed during the development of pathological cardiac hypertrophy, such as atrial natriuretic peptide (*Nppa*)^24^, brain natriuretic peptide (*Nppb*)^24^, regulator of calcineurin 1 (*Rcan1*)^25^ and the fibrosis marker collagen type I alpha (*Col1-α*)^26^. Results show that *Nppb* expression was significantly increased in the KO mice while the other three genes showed a slight, although not significant, increment (Supplementary Table I). Cardiac hypertrophy is a phenotypic change in response to a physiological or pathological stimulus^1^; in pathological cases, an irreversible tissue remodeling occurs, gradually leading to heart failure. To characterize the myocardial function of Herpud1-KO mice, various echocardiographic parameters were measured in M-mode (Fig. 3a). The Herpud1-KO animals showed significant decreases in fractional shortening (FS), ejection fraction (EF), and stroke volume (Fig. 3b–d) as compared to WT mice, confirming the importance of Herpud1 in myocardial function. Additionally, both left-ventricular end-systolic diameter (LVESd) and volume (LVESv) were significantly higher in Herpud1-KO mice as compared to their WT counterparts, while left-ventricular end-systolic parameters (LVEDd and LVEDv) showed no significant between-group differences (Table 1). The latter finding suggests that the left ventricular cavity is not affected in Herpud1-KO mice during relaxation but does exhibit impaired contractile capacity during systole, reflected as a reduced ejection volume. Taken together, the results show that a Herpud1 deficiency leads to the development of cardiac hypertrophy and impaired myocardial function.

**Table 1:** Systolic function of Herpud1-KO mice.

Parameter	WT	Herpud1 KO	*P value*
LVESd (mm)	2.76 ± 0.04	2.94 ± 0.04	0.011*
LVEDd (mm)	4.01 ± 0.04	4.03 ± 0.06	0.866
LVESv (µL)	28.79 ± 1.17	33.37 ± 1.19	0.012*
LVEDv (µL)	70.80 ± 1.81	71.50 ± 2.83	0.84

LVESd: Left ventricle end systole diameter (mm).

LVEDd: Left ventricle end diastole diameter (mm).

LVESv: Left ventricle end systole volume (µL).

LVEDv: Left ventricle end diastole volume (μL).

Mean ± SEM analyzed by *t*-test (n = 12). *P < 0.05 vs. WT.

### *In vitro* Herpud1 deficiency induces cardiomyocyte hypertrophy

**Figure 4:**
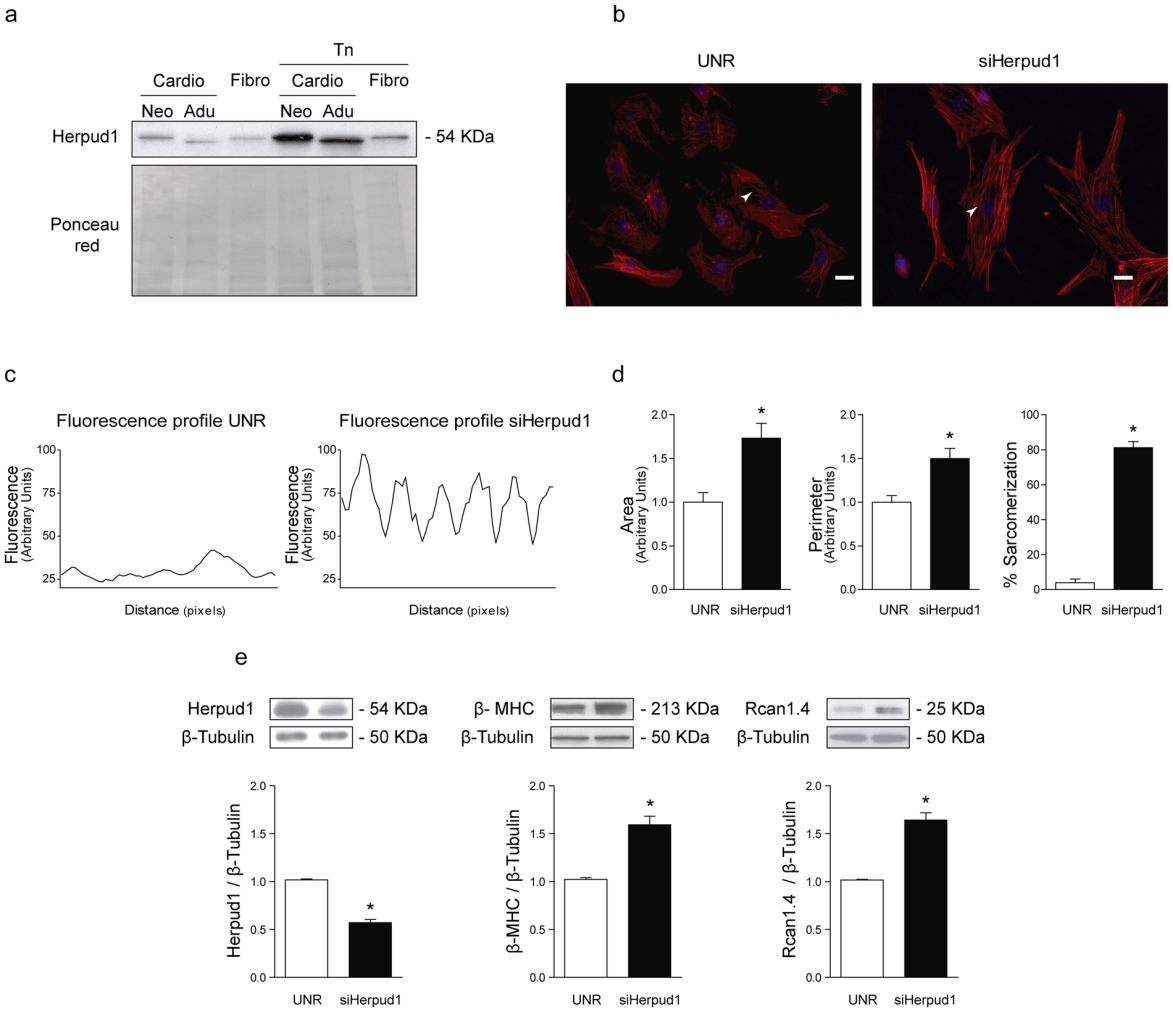
Herpud1 siRNA triggers cardiomyocyte hypertrophy. (**a**) Representative Western blot of Herpud1 protein levels in isolated neonatal (Neo) and adult (Adu) cardiomyocytes (Cardio) and neonatal fibroblasts (Fibro) cultured under basal conditions or treated with tunicamycin for 4 h (Tn, 1.2 mM). Ponceau red shows the loading of each sample (n = 3). (**b**) Representative images (400X) of fixed NRVM stained with the fluorescent probe rhodamine-phalloidin after incubation with unrelated siRNA (UNR, 200 nM) or anti-Herpud1 siRNA (siHerpud1 #1) (200 nM) for 48 h (n = 3). (**c**) White arrows show analyzed sarcomeres in the fluorescent profiles of sarcomeres from the previous images. (**d**) Results for area, perimeter, and percentage of sarcomerized cells for fixed NRVM stained with rhodamine-phalloidin after incubation with unrelated siRNA (UNR) or anti-Herpud1 siRNA (siHerpud1 #1) for 48 h (n = 3). (**e**) Representative Western blots for Herpud1 (n = 6), β-MHC (n = 6), and Rcan1.4 (n = 4) for NRVM incubated with unrelated siRNA (UNR) or anti- Herpud1 siRNA (siHerpud1#2) for 48 h. Graphs represent the measurements for each protein using β-tubulin as the loading control. Mean ± SEM, analyzed using a *t*-test. *p < 0.05 vs. UNR.

We assessed the presence of the Herpud1 protein in the two main cardiac cell types, cardiomyocytes and fibroblasts, under basal and ER stress-induced conditions, where Herpud1 overexpression has been widely documented^17,27^. Neonatal rat ventricular myocytes (NRVM) and cardiac fibroblasts and adult rat cardiomyocytes were used in this analysis. Herpud1 was present in all three cell types and overexpressed after treatment with the ER stressor tunicamycin^28^ (Fig. 4a). To assess the effect of Herpud1 silencing on cardiomyocytes, NRVM were transfected with one of two different Herpud1 siRNAs (#1 and #2). In both cases, Herpud1 protein levels were decreased to approximately 50% after 48 h with a siRNA concentration of 200 nM (Supplementary Fig. 3). The same conditions were used in all subsequent Herpud1 knockdown experiments. Morphological studies were also performed in Herpud1-knockdown NRVM. F-actin was stained with rhodamine-phalloidin (Fig. 4b), allowing for quantification of the cell area, cell perimeter, and percentage of sarcomerized cells, defined as cells with an ordered, stair-like fluorescence pattern (Fig. 4c). Results show that Herpud1 knockdown induced significant increases in cell area, perimeter and sarcomerization in cardiomyocytes (Fig. 4d). In addition, the levels of hypertrophy markers beta-myosin heavy chain (β-MHC) and calcineurin regulator 1.4 (Rcan 1.4) were assessed in Herpud1-silenced NRVM. β-MHC is considered a general marker of hypertrophy^5^, and Rcan1.4 is a physiological regulator of calcineurin whose expression is induced by a Ca^2+^-dependent calcineurin activation, thus accounting for the activation of the pro-pathological hypertrophic Ca^2+^/calcineurin/NFAT axis^4^. Both β-MHC and Rcan1.4 levels were increased in Herpud1-knockdown NRVM (Fig. 4e). Taken together, the above results suggest that reduced Herpud1 protein levels alone are sufficient to trigger an intrinsic hypertrophic phenotype in NRVM, with pathological features such as elevated Rcan 1.4 levels.

### Herpud1 deficiency leads to decreased IP3R degradation and increased intracellular calcium

**Figure 5:**
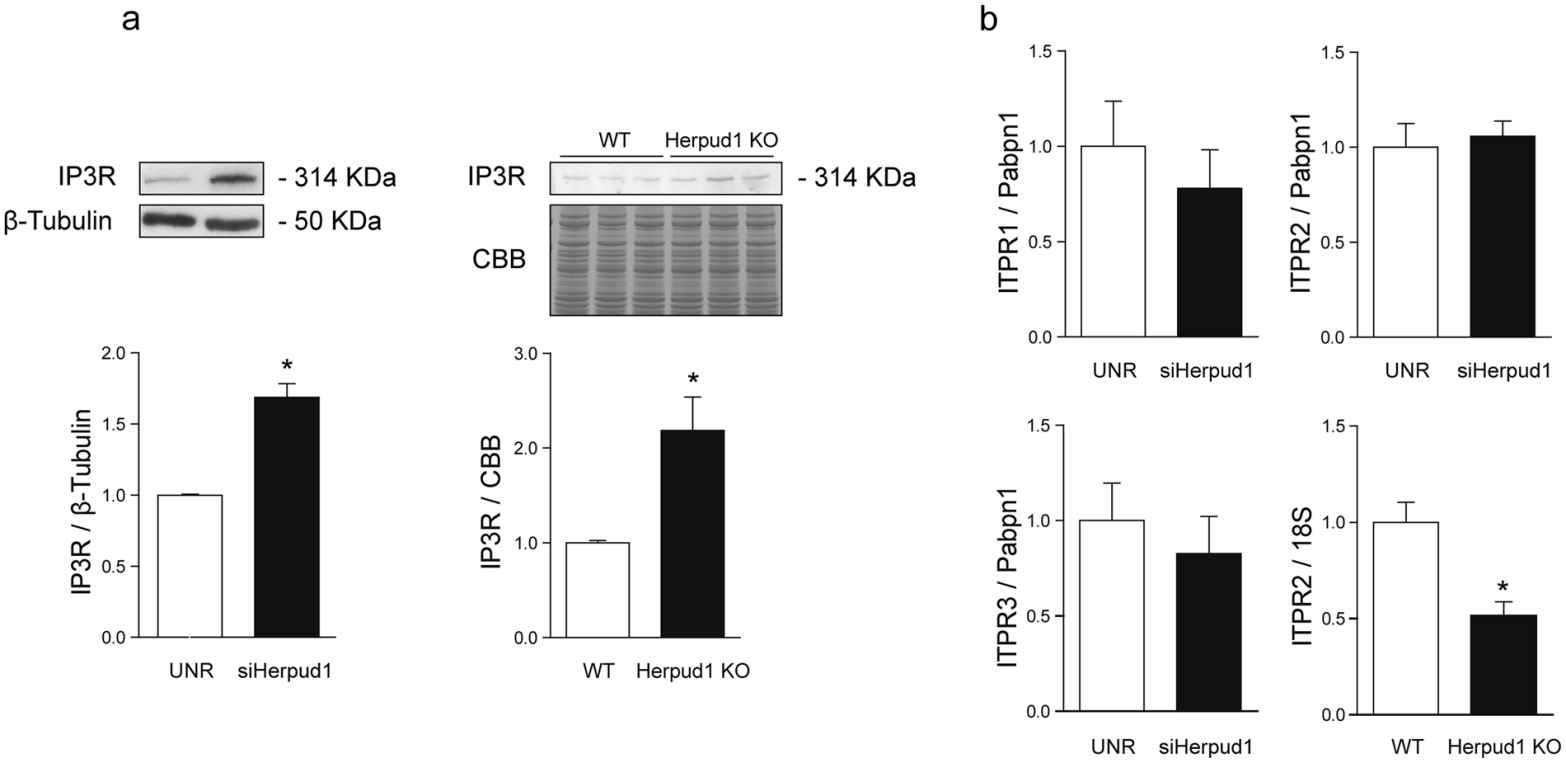
IP3R protein and mRNA levels in cardiomyocytes treated with Herpud1 siRNA and in hearts from Herpud1-KO mice. (**a**) Representative Western blot of IP3R protein levels in *Left:* control (UNR) and Herpud1-knockdown NRVM (siHerpud1 #2, 48 h) (n = 6) and *Right:* samples from wild-type (WT) and Herpud1-knockout (Herpud1 KO) mice (n = 3). (**b**) Graphs show the relative expression of IP3R1, IP3R2, and IP3R3 mRNA in control (UNR) and Herpud1-knockdown NRVM (siHerpud1 #2, for 48 h) (n = 4) and IP3R2 mRNA in cardiac samples from wild-type (WT) and Herpud1-knockout (Herpud1 KO) 10-to-12-week-old mice (n = 3). Pabpn1 was used as a housekeeping messenger in isolated NRVM, and the 18 S ribosomal subunit was used in the cardiac tissue samples. Mean ± SEM, analyzed using a *t*-test. *p < 0.05 vs. UNR.

**Figure 6:**
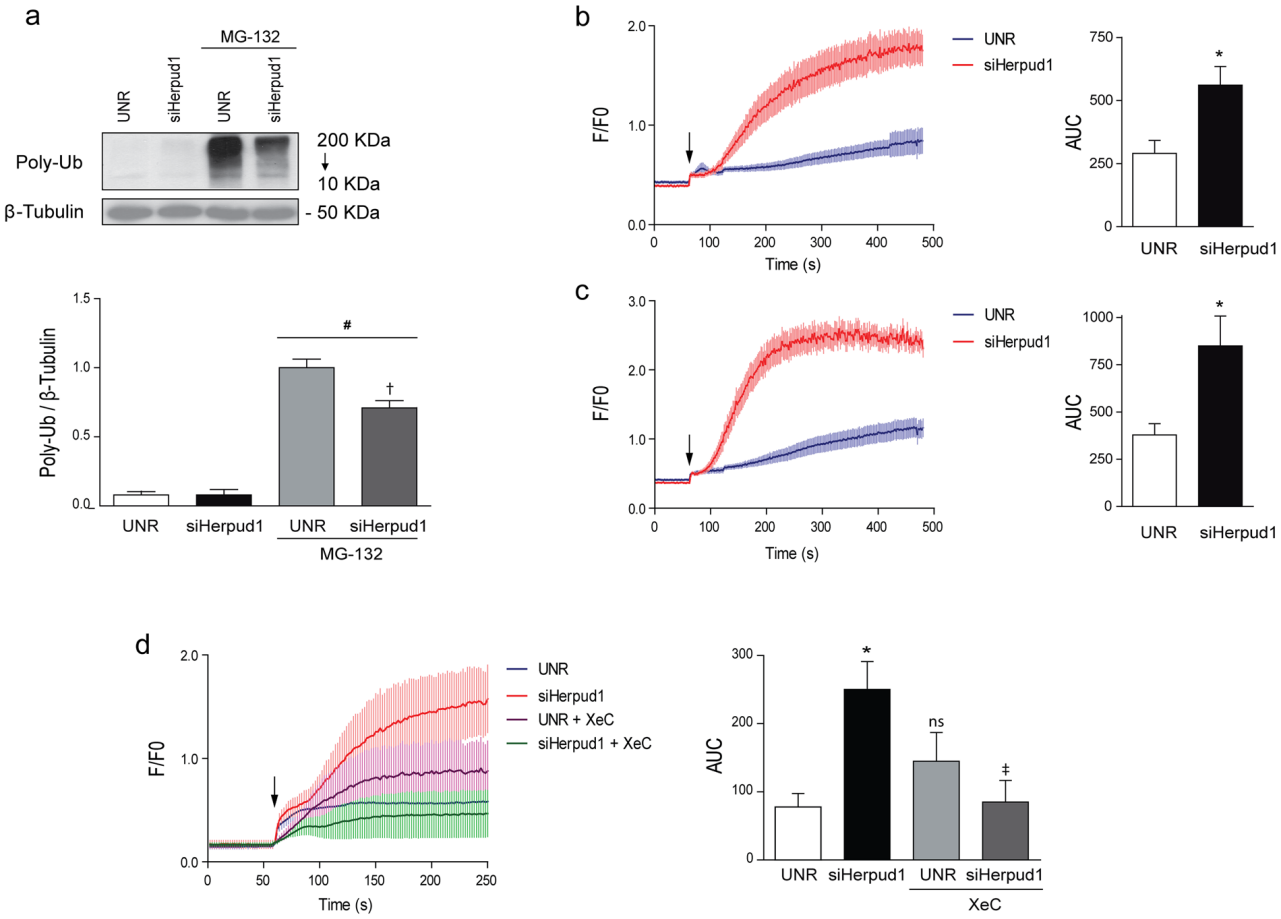
Effect of Herpud1 siRNA on polyubiquitin levels and cytosolic and nuclear Ca^2+^ levels in cultured NRVM. (**a**) Representative Western blots for lysine 48-linked polyubiquitin (Poly-Ub) in control (UNR) and Herpud1-knockdown NRVM (siHerpud1 #2, 200 nM, 48 h) in the presence or absence of the proteasome inhibitor MG-132 (4 μM, 24 h). Graphs correspond to the quantification of each lane, using β-tubulin as a loading control (n = 6). (**b**) Graph of cytosolic Ca^2+^ concentration kinetics and quantification of the area under the curve (n = 3). (**c**) Graph of nuclear Ca^2+^ kinetics and quantification of the area under the curve (n = 3). Both (b) and (c) correspond to control (UNR) and Herpud1-knockdown NRVM (siHerpud1 #2, 48 h). (**d**) Graph of cytosolic Ca^2+^ concentration kinetics and quantification of the area under the curve, in control (UNR) and Herpud1-knockdown NRVM (siHerpud1 #2, 48 h) in the absence or presence of xestospongin C (XeC, 100 μM, 30 min) (n = 3). The black arrow shows the time of addition of histamine (100 mM). Mean ± SEM analyzed using a *t*-test or one-way ANOVA followed by Bonferroni’s post-test. *p < 0.05 vs. UNR, ^†^ *p* < 0.05 vs. UNR + MG-132; ^#^ *p* < 0.05 vs. same condition without MG-132, ^‡^p < 0.05 vs siHerpud1.

Ca^2+^ is a key second messenger in the generation of cardiac hypertrophy^10^. The release of Ca^2+^ from the ER into the cytosol or nucleus plays a major role in the activation of pro-hypertrophic pathways^29^. Therefore, IP3Rs may be of paramount relevance in the Ca^2+^ signaling that leads to cardiac hypertrophy. IP3R degradation occurs through the proteasome^22^, and the degradation of isoform 1 depends on Herpud1 in PC12 cells^20^. To investigate whether this dependence is also observed in cardiac tissue, IP3R levels were evaluated in Herpud1-knockdown NRVM and cardiac samples from Herpud1-KO mice. IP3R protein levels were elevated in both models as compared to control conditions (Fig. 5a). This increase could be attributed to two different processes: increased IP3R synthesis and/or decreased IP3R degradation. To shed more light on this process, therefore, IP3R mRNA content was quantified in the samples. Despite the increased IP3R protein levels observed, the Herpud1-knockdown NRVM did not show significantly increased mRNA levels for any of the IP3R isoforms. In fact, decreased levels of isoform 2 transcripts were seen in Herpud1-KO cardiac tissue as compared to WT tissue (Fig. 5b). Taken together, these results suggest that the changes in IP3R content associated with a Herpud1 deficiency are likely attributable to decreased IP3R degradation rather than increased *de novo* synthesis of IP3R. These results also suggest that Herpud1 may participate in IP3R degradation, as Herpud1 has known degradative functions^30^. For instance, Herpud1 ubiquitin-like domain (ULD) allows for the polyubiquitination of ER proteins, driving them towards degradation^30^. Since no reports have addressed this potential function of Herpud1 in cardiomyocytes, the proteasomal degradation pathway was analyzed in Herpud1-knockdown NRVM. Lysine 48-linked polyubiquitin content was measured in the presence and absence of MG-132, an inhibitor of proteasomal activity. Levels of polyubiquitin aggregates were significantly lower in MG-132-treated cells after Herpud1 silencing (Fig. 6a), suggesting that Herpud1 indeed participates in the polyubiquitination of a relevant number of proteins in cardiomyocytes, possibly including IP3R.

Herpud1 has also been proposed as a regulator of Ca^2+^ based on its capacity to modulate the release of this cation from the ER^20,21^. As mentioned, it remains unknown whether Herpud1 exercises this function in cardiomyocytes, where Ca^2+^ signaling is crucial to a wide variety of intracellular processes including contraction, signal transduction, hypertrophy development, electrical signaling, gene transcription, and cell death^31^. To evaluate whether the observed increase in IP3R protein levels also drives an increase in cytoplasmic Ca^2+^, cytoplasmic and nuclear Ca^2+^ levels were measured, using the ratiometric fluorescent probe Fura-2AM. Ca^2+^ levels were measured in the presence of histamine, which induces the generation of IP3 and the release of Ca^2+^ from the ER through IP3R activation, and in the absence of external Ca^2+^. Both cytoplasmic (Fig. 6b) and nuclear (Fig. 6c) Ca^2+^ levels were elevated in Herpud1-silenced NRVM as compared to control cells. An increase in cytosolic Ca^2+^was also seen in NRVM preincubated with MG-132 (1 μM for 3 h, Supplementary Fig. 4). Interestingly, the IP3R antagonist xestospongin C (XeC. 100 μM for 30 min, Fig. 6d) prevents histamine-induced Ca^2+^ signal in Herpud1-knockdown NRVM. This result supports the notion that the increased level of IP3R protein observed upon Herpud1 silencing stimulates the release of Ca^2+^ from the ER into other intracellular compartments.

## Discussion

Diverse proteins and cellular processes are involved in the genesis and progression of pathological cardiac hypertrophy. In the present study, we evaluated the potential of Herpud1 as a novel negative regulator of cardiomyocyte hypertrophy and therefore as a putative new pharmacological target.

**Figure 7:**
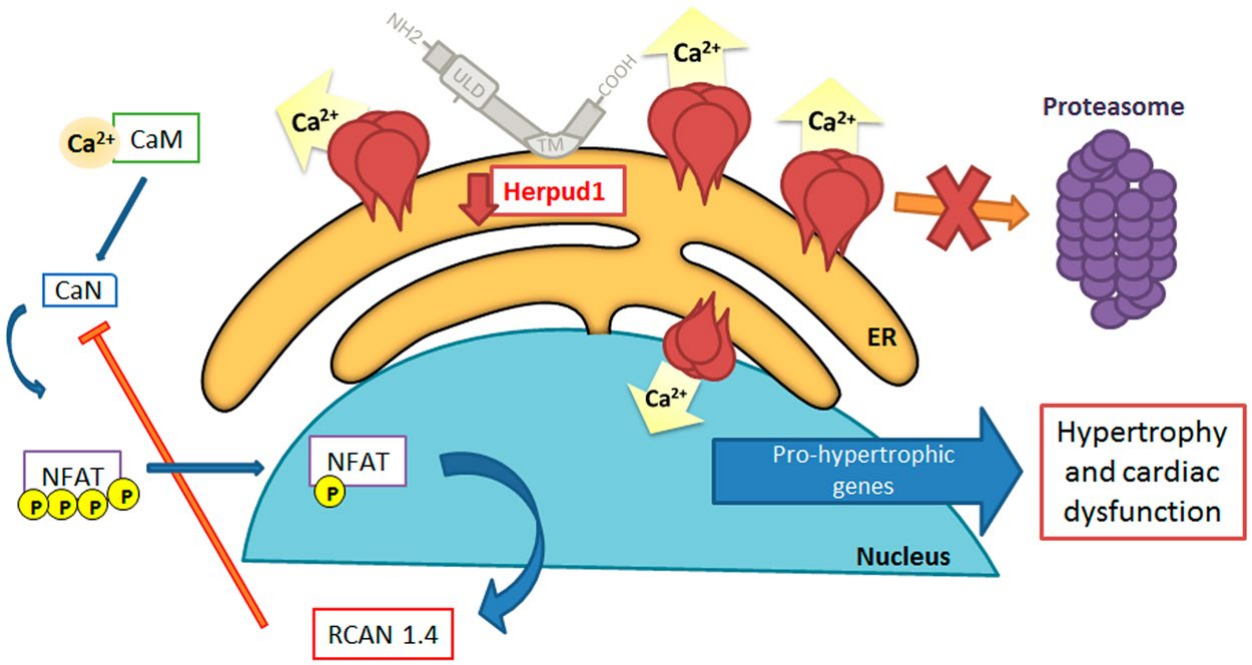
Proposed model for hypertrophy induced by Herpud1 silencing. Herpud1 plays a role in the regulation of Ca^2+^ levels in cardiomyocytes, possibly by modulating IP3R degradation. Reduction in Herpud1 protein levels allows for activation of pro-hypertrophic signaling cascades such as the calcineurin/NFAT pathway. This new evidence suggests an alternative model for the development of pathological cardiac hypertrophy and a novel putative pharmacological treatment target.

Our results show for the first time that the Herpud1 protein is present in the heart, specifically in cardiomyocytes and fibroblasts. Deletion or reduction of this protein induces cardiac hypertrophy both *in vivo* and *in vitro*, indicating the importance of Herpud1 in maintaining basal homeostasis as well as suggesting a potential role in anti-hypertrophic mechanisms. Furthermore, Herpud1-knockout mice suffered a significant decrease in myocardial function, including decreased ejection fraction, fractional shortening, and stroke volume. We also found that IP3R protein levels were elevated and that Ca^2+^ release from intracellular reservoirs was increased when this receptor was activated by histamine, a phenomenon that may facilitate activation of pro-hypertrophic pathways. We propose a new model for the generation of pathological hypertrophy *in vivo* and *in vitro* based on our findings of decreased cardiac contractile function in isolated NRVM, as well as increased protein levels for a negative regulator of calcineurin (Fig. 7).

Because complete Herpud1-knockout mice were used in the present study, it is difficult to rule out the participation of other organs or extra-cardiac processes, such as increased blood pressure or renal failure, in triggering the observed hypertrophic phenotype. However, the hypertrophic phenotype and the loss of cardiac function observed *in vivo* was correlated with the cardiomyocyte hypertrophy observed upon Herpud1 silencing *in vitro*, supporting the contention that the pathological phenomenon is likely caused by an intrinsic process of the contractile cells. Moreover, while it has been widely described that pressure or volume overload triggers left ventricular hypertrophy^32^ and that increased pulmonary pressure causes right ventricular hypertrophy^33^, there was no specific increase in right or left ventricular weight (Supplementary Table 2), although there was a general increase in organ size involving both the atria and ventricles and a significant increase in cardiomyocyte cross-sectional area (Fig. 2). Furthermore, it would seem that the process triggered by Herpud1 silencing is not attributable to hemodynamic changes or increased sympathetic tone as there were no significant differences in heart rate between WT and Herpud1-knockout mice (Supplementary Table 2), although the latter group developed cardiac hypertrophy and dysfunction. Finally, it has been described that cardiac hypertrophy accompanied by decreased ejection fraction is evidence of intrinsic cardiomyocyte dysfunction, which is associated with extrinsic heart growth and therefore increased risk of progression into heart failure^34^. However, the long-term effects of the phenotypic change observed in the knockout mice, such as increased prevalence of heart failure at older ages, remain unknown.

The activity of Ca^2+^-dependent signaling pathways such as calcineurin/NFAT increases during the progression to pathological hypertrophy, and the perpetuation of these signals depends on overexpression of IP3R^12^. Evidence regarding the role of these pathways during later stages of heart failure is controversial, however, as some reports suggest that IP3R and other Ca^2+^-handling proteins are downregulated at these stages^35^, while others have shown a redistribution of ryanodine receptor type 2 and sarco/endoplasmic reticulum Ca^2+^-ATPase type 2a as well as increased IP3R2 levels^14^. In the present work, the IP3R protein levels increased during the hypertrophy induced by Herpud1 silencing, possibly triggering a Ca^2+^-dependent induction of the hypertrophic phenotype. However, the Herpud1-knockout mice and knockdown cells, respectively, showed decreased or unchanged cardiac IP3R mRNA levels, indicating that the increased IP3R levels were due to decreased degradation rather than increased IP3R transcription.

As mentioned above, Herpud1 participates in the degradation of ER proteins through the proteasome, facilitating their ubiquitination and transport^20,30^. Therefore, lysine 48-linked polyubiquitin aggregates were measured in control and Herpud1-silenced NRVM to confirm the degradative function of the latter protein in cardiac cells. In agreement with previous reports^18–20^, our results showed that Herpud1 mediates the proteasomal degradation of a significant number of proteins, possibly including IP3R, as its silencing significantly decreased the polyubiquitin aggregate content.

One of the questions arising from this work is how the hypertrophic process is triggered under basal conditions, without exogenous stimuli such as neurohumoral factors and/or mechanical stress. Even if IP3R protein levels were elevated due to a partial or complete absence of Herpud1, intracellular Ca^2+^ levels would not necessarily increase, since IP3 must bind to its receptor to allow for channel opening. Therefore, it remains unclear how the increase in Ca^2+^ and the hypertrophic signaling are triggered. It is important to note that a cell-signaling pathway has a basal level and is gradually activated. In the present case, the pathway may be triggered to induce the hypertrophic phenotype by increased IP3R protein levels. Each of the three IP3R isoforms has a different sensitivity to IP3-induced activation and Ca^2+^-induced deactivation^36–38^. Isoform 2, which is the isoform primarily expressed in cardiac tissue, may be activated by basal IP3 concentrations, and its deactivation may require higher Ca^2+^ concentrations than the other isoforms^15,39^. As a result, cardiomyocytes have a more sensitive and complex Ca^2+^ signaling profile than other cell types. Therefore, it is possible that elevated IP3R protein levels alone may trigger pathological hypertrophy in cardiomyocytes, both in isolated cells and whole hearts. However, because the IP3R antibody used in this study recognizes all three isoforms, a future study to measure the protein levels of each variant may shed more light on the role of IP3R2 in the Herpud1-dependent hypertrophic process.

Diverse evidence suggests that intracellular Ca^2+^ is a critical signal for the development of cardiac hypertrophy^10^. The two major pathways linked to the genesis of cardiac hypertrophy, calcineurin/NFAT and histone deacetylase, are Ca^2+^-dependent^4,40^. As mentioned before, elevated cytosolic and nuclear levels of Ca^2+^ were observed in Herpud1-knockdown NRVM (Fig. 6b–c). A similar effect was seen upon the addition of the proteasomal inhibitor MG-132 (Supplementary Fig. 4). This effect may be associated with the ability of Herpud1 to regulate the degradation of proteins involved in the Ca^2+^ release from the ER, as shown in other cellular models^20,21,41^. Furthermore, the increment on cytosolic Ca^2+^ levels induced by histamine in Herpud1-knockdown was completely inhibited by the antagonist of IP3R XeC (Fig. 6d). These results support the notion that the Ca^2+^ increasing effect of the silencing of Herpud1 is caused by an increase in the levels of IP3R. The development of cardiac hypertrophy also depends on nuclear Ca^2+^ signaling mediated by activation of IP3R in the nucleosome^14^. This Ca^2+^ release plays a key role in the regulation of gene expression. The increased nuclear levels of Ca^2+^ facilitate a rapid change in gene expression that has been linked to cardiomyocyte hypertrophy^42^. This rapid response may underlie the phenotypic change observed in Herpud1-knockout mice and NRVM treated with Herpud1 siRNA. Since the latter experiments were performed in the absence of external Ca^2+^, intracellular reservoirs are likely the source of the increased cytosolic and nuclear Ca^2+^. Herpud1 is apparently capable of regulating the ER Ca^2+^ pool, as Chan *et al*.^41^ showed that Herpud1 overexpression lowers the amount of Ca^2+^ released by the ER after thapsigargin treatment. Therefore, our results could be explained by an increased availability of Ca^2+^ channels and/or a larger intracellular Ca^2+^ pool.

Some limitations of the present study are: a) The evaluation of heterozygous littermates as well as the time course of myocardial function of Herpud1 KO mice over time were not done and future work should study these points. b) Immunohistochemistry of Herpud1 shows some non-specific immunoreactivity in Herpud1 KO mice (Supplementary Fig. 2), in agreement with the detection of a non-specific band in Western blot.

In summary, our data show that Herpud1 plays a significant role in the regulation of Ca^2+^ levels, possibly by modulating IP3R degradation, and that reducing Herpud1 protein levels allows for activation of a pro-hypertrophic signaling pathway (Fig. 7). These findings also suggest an alternative model for the development of pathological cardiac hypertrophy and a novel putative pharmacological target, as increasing Herpud1 levels may prevent or revert pathological cardiac hypertrophy and myocardial dysfunction.

## Methods

### Antibodies and reagents

The following reagents were purchased from Sigma-Aldrich: Hank’s medium, DME medium (DMEM), 199 medium (M199), pancreatin, gelatin, Triton X-100, 5-bromo-2′-deoxiuridine, anti-β-tubulin and anti-Rcan1 antibodies, and Herpud1, Mission #1 (universal negative control) siRNAs and Xestospongin C. The fluorescent probe Fura-2 AM, paraformaldehyde, and Hoechst 33342 were obtained from Thermo Fisher Scientific. The proteasome inhibitor MG-132 was purchased from Merck Millipore. Tunicamycin and the anti-Herpud1 antibody were from Enzo Life Science. Anti-IP3R antibody was purchased from Abcam. Anti-polyubiquitin Lys48 from Cell Signaling Technology and anti-β-MHC from Vector Laboratories. Secondary rabbit and rat anti-IgG antibodies conjugated with peroxidase were from Calbiochem. ECL chemiluminescent reagent for Western blotting was purchased from Amersham BioSciences. All SDS-PAGE materials and PVDF membranes were from Bio-Rad Laboratories. The mounting medium for fluorescence was purchased from DAKO Corporation. All other organic and inorganic reagents, salts, acids, and solvents were purchased from Merck unless otherwise specified.

### Experimental models

Primary neonatal rat ventricular myocytes (NRVM) or adult rat cardiomyocytes and cardiac fibroblasts were obtained from 1-to-3-day-old and 2-month-old Sprague-Dawley rats, respectively (Animal Facility, Facultad de Ciencias Químicas y Farmacéuticas, Universidad de Chile, Santiago). Genetically-modified Herpud1-knockout mice were obtained from the National Cerebral and Cardiovascular Center, Osaka, Japan. All animals were handled according to the Guide for the Care and Use of Laboratory Animals (NIH, 2011), and the experimental protocols were approved by the Bioethics Committee of the Faculty of Chemical and Pharmaceutical Sciences, University of Chile, Santiago (#BE2014-01).

### Cardiomyocyte culture

NRVM were prepared as described previously^43^. Cells were maintained in DMEM/M199 (4:1) medium. Cells were plated at a density of 1–8 × 10^3^ mm^−2^ on gelatin-coated 12-well plates or 35- or 60-mm Petri dishes. For Ca^2+^ and immunofluorescence studies, NRVM were plated on gelatin-coated 25- and 18-mm glass coverslips in 35-mm or 12-well Petri dishes, respectively.

### Cardiomyocyte transfection

Small interfering RNAs (siRNA) for Herpud1 and a negative control (Mission #1, SIC001, Sigma-Aldrich) were used according to manufacturer instructions. Cells were grown and transfected with siRNAs at a concentration of 100-200 nM with Oligofectamine™ (Invitrogen): Herpud1 #1: SASI_RN01_00185592 or Herpud1 #2: SASI_RN01_00057861. After 6 h of incubation in Opti-MEM®, the medium was removed and replaced with DMEM/M199. Downregulation of Herpud1was assessed 48 h post-transfection.

### Herpud1-KO mice

These mice were generated in the laboratory of Dr. Koichi Kokame (National Cerebral and Cardiovascular Center, Osaka, Japan). Full Herpud1-KO C57BL/6 mice were generated by crossing C57BL/6 wild-type mice with Herpud1^+/−^ mice for 10 generations. Heterozygous mice were obtained from 129/SvJ-derived Go Germline embryonic stem cells (Incyte Genomics). Herpud1-KO mice showed normal development with no apparent phenotypic abnormalities under normal breeding conditions, as described in Eura *et al*.^23^. All animals used were 10-to-12-week-old males. Following ultrasound measurements, mice were sacrificed and weighed, and samples from the heart, liver, skeletal muscle, and brain were obtained for further analysis. One tibia was also extracted from each mouse and subjected to digestion with 2 mg mL^−1^ proteinase K (Sigma-Aldrich) in lysis buffer (50 mM Tris, 100 mM EDTA, 100 mM NaCl, 1% SDS) at 55 °C overnight to clean the bones of cartilage and muscle. Once clean, tibia length was measured for each mouse.

### PCR genotyping

The Herpud1-KO mice were identified using PCR genotyping of genomic DNA samples obtained from an ear. The primers used are described below, and the amplification products (94 °C for 30 s, 60 °C for 30 s, and 72 °C for 30 s; 30 cycles) were separated by agarose gel electrophoresis and stained with SYBR Green/Nucleic acid gel stain (Lonza). Bands were detected using a LAS-3000 Image Analyzer (Fujifilm) equipment. Primers used were: *Herpud1* (WT) F: 5′-CCCCTCCCCCTTTGGTTGACA-3′ R: 5′-TCCAGGGGCTTAGACGCTTAC-3′ 343 bp and Herpud1* (KO) F: 5′-CCCCTCCCCCTTTGGTTGACA-3′ R: 5′-TGGACCTGGGAGTGGACACCT-3′ 252 bp.

### Western blot analysis

Equal amounts of cell protein extracts were separated by SDS-PAGE (8–15% polyacrylamide gels) and electrotransferred to PVDF membranes. Membranes were blocked with 5% milk in Tris-buffered (pH 7.6), containing 0.1% (v/v) Tween 20 (TBST). Membranes were incubated with primary antibodies at 4 °C and re-incubated with horseradish peroxidase-linked secondary antibody [1:5,000 in 1% (w/v) milk in TBST]. The bands were detected using ECL with exposure to Kodak film or a LAS-3000 Image Analyzer (Fujifilm) and quantified with scanning densitometry. Protein content was normalized to β-tubulin or total protein content as measured using Ponceau red or Coomassie brilliant blue staining (CBB).

### Histological and immunocytochemical studies

Hearts from both WT and Herpud1 KO mice were fixed in 4% paraformaldehyde and transferred to 1x PBS, followed by paraffin embedding. Hematoxylin/Eosin (H&E) staining was performed for morphological analyses. Wheat germ agglutinin (WGA) was used for cross-sectional area measurements, quantified from at least 50 cells per group as described by Pedrozo *et al*.^46^. Anti Herpud-1 rabbit polyclonal Thermo Fisher (Pa5-29469, 1:200 pH 8.0) was used for immunohistochemistry and immunocytochemistry assays.

### Echocardiography

Mice were initially anesthetized in a chamber with isoflurane (3.5%) and then kept under isoflurane anesthesia (1.7%) in a 1:1 air-oxygen mixture. During the procedure, body temperature and heart rate were monitored, establishing 36 °C and 400 beats per min, respectively, as inferior limits. Vevo2100 equipment from PrimeTech with a MicroScan™ Transducer (MS-550) was used. The corresponding images were captured in M-mode and analyzed using the equipment software. All measurements were performed on the papillary muscles.

### RNA purification and real-time reverse transcription PCR (RT-PCR)

Total RNA was isolated using TRIzol according to the manufacturer protocol (Invitrogen). Random hexamers were used for reverse transcription reactions with Superscript II (Invitrogen). RT-PCR was performed using PowerUp SYBR Green Master Mix, and the results were analyzed using the Step One^TM^ (Thermo Fisher Scientific) system. The primers used were:

*Itpr1* (rat) F: 5′-GAGAGAAAGCGCACGCCGAGA-3′ R: 5′-GCAATCCCATGTCCGCGAAGAG-3′; *Itpr2* (rat) F: 5′-TCTTGGTGGATATGGCAAGGGTCT-3′ R: 5′-AGTTGAAGAAGCCGCTCACGATGT-3′; *Itpr3* (rat) F: 5′-CAGAACGACCGCAGGTTTGTCAT-3′ R: 5′-TTCTGCCCATTGTTGGGAACATCG-3′; *Nppa* (mouse) F: 5′-CTTCTTCCTCGTCTTGGCCT-3′ R: 5′-CTGCTTCCTCAGTCTGCTCA-3′; *Nppb* (mouse) F: 5′-CATGGATCTCCTGAAGGTGC-3′ R: 5′-CCTTCAAGAGCTGTCTCTGG-3′; *Rcan1* (mouse) F: 5′-CCCGTGAAAAAGCAGAATGC-3′ R: 5′-TCCTTGTCATATGTTCTGAAGAGGG-3′; *Col1- α:* (mouse) F: 5′CTGACGCATGGCCAAGAAGA-3′ R: 5′-TACCTCGGGTTTCCACGTCT-3′; *Pabpn1* (rat) F: 5′-GTTGGCAATGTGGACTATGG-3′ R: 5′-AACAGGGACTCATCTAAGGC-3′; *18S* (mouse) F: 5′-CGGACAGGATTGACAGATTG-3′ R: 5′-CAAATCGCTCCACCAACTAA-3′. Additionally, one-step quantitative reverse transcription was then performed on the tissue samples using the One-Step RT-PCR Kit (Qiagen). The catalog numbers for the primers (included in the kit) were as follows: QF00503041 for Herpud1, QF00196504 for ITPR2, and QF00289576 for 18S. Data for each transcript were normalized to Pabpn1 or 18S mRNA as an internal control using the 2−ΔΔCt method to calculate relative transcript abundances.

### Assessment of cardiomyocyte hypertrophy

Area, perimeter, percent sarcomerization, and sarcomere fluorescence profiles were assessed using epifluorescence microscopy (Carl Zeiss Axiovert 135, LSM Microsystems) in paraformaldehyde-fixed cells permeabilized with Triton X-100 and treated with Hoechst 33342 (to stain nuclei) and rhodamine-phalloidin (to stain F-actin) at a 1:500 dilution (Thermo Fisher Scientific), as previously described^44^. At least 50 cells from randomly-selected fields were analyzed using ImageJ software (NIH).

### Intracellular Ca^2+^ measurements

Intracellular Ca^2+^ signals were determined as described previously^45^. Images were obtained from cultured cardiomyocytes preloaded with Fura-2AM (Thermo Fisher Scientific) using a Spinning Disk IX81 confocal microscope (Olympus). Coverslips were mounted in a 1 mL capacity chamber and placed in the microscope for fluorescence measurements after excitation with a laser line of 340/380 nm. In some selected experiments, the cells were preincubated with MG-132 (1 μM for 3 h)^46^ or with XeC (100 μM for 30 min)^47^. In these experiments, a pulse of histamine (Sigma-Aldrich) was added after 60 s at a final concentration of 100 mM to increase Ca^2+^ movement from the ER to cytosol via IP3R^47^. The fluorescent images were collected every 2 s and analyzed frame-by-frame with ImageJ software (NIH).

### Statistical analysis

Results are shown as representative images or mean ± SEM of at least three independent experiments. All data had a normal distribution, and differences were analyzed using Student’s *t*-test or ANOVA with a Bonferroni’s post-test. *p* < 0.05 was the threshold for statistical significance.

## Electronic supplementary material

Completed Dataset

Supplemental Material
