# Supplementary material for: Herpud1 negatively regulates pathological cardiac hypertrophy by inducing IP3 receptor degradation
Source: Sci Rep. 2017 Oct 17;7:13402. doi: 10.1038/s41598-017-13797-z (PMC5645377; doi:10.1038/s41598-017-13797-z)
Supplement: Supplementary file 2 — Supplemental Material [file 41598_2017_13797_MOESM2_ESM.pdf]

SREP-17-25760-T-R1

Herpud1 negatively regulates pathological cardiac hypertrophy by inducing IP3 receptor degradation

Natalia Torrealba[1], Mario Navarro-Marquez[1], Valeria Garrido[1], Zully Pedrozo[1,2], Diego Romero[3], Yuka Eura[4], Elisa Villalobos[1,5], Juan Carlos Roa[3], Mario Chiong[1], Koichi Kokame[4,*] & Sergio Lavandero[1,2,5*]

[1] *Advanced Center for Chronic Disease (ACCDiS) & Center for Molecular Studies of the Cell (CEMC), Facultad de Ciencias Químicas y Farmacéuticas & Facultad de Medicina*
[2] *Instituto de Ciencias Biomédicas, Facultad de Medicina Universidad de Chile, Santiago, Chile*
[3] *Department of Pathology, Advanced Center for Chronic Diseases (ACCDiS), Faculty of Medicine, Pontifical Catholic University of Chile, Santiago, Chile*
[4] *Department of Molecular Pathogenesis, National Cerebral and Cardiovascular Center, Suita, Osaka, Japan*
[5] *Department of Internal Medicine (Cardiology Division), University of Texas Southwestern Medical Center, Dallas, Texas, USA*

Running title*:* Herpud1 negatively regulates cardiac hypertrophy

Keywords: Herpud1, Herp, IP3 receptor, Heart, Hypertrophy, Protein degradation

*Correspondence and requests for materials should be addressed to K.K. (kame@ncvc.go.jp) or S.L. (slavander@uchile.cl).

Supplementary Information

Supplementary Table 1 I Relative expression of mRNA in mice

**Gene**	**WT**	**Herpud1 KO**	***P* value**
Herpud1	1.00 ± 0.37	2.2E-05 ± 1E-05	<0.0001*
Nppa	1.00 ± 0.22	1.38 ± 0.39	0.44
Nppb	1.00 ± 0.09	1.52 ± 0.10	<0.05*
Rcan1	1.00 ± 0.12	2.35 ± 0.54	0.07
Col1-α	1.00 ± 0.05	1.19 ± 0.17	0.36

18S ribosomal subunit was used as housekeeping messenger.

Mean ± SEM analyzed by *t*-test (n=3). **P* value vs. WT

Supplementary Table 2 I Mouse measurements

Parameter	WT	Herpud1 KO	*P* value
Body weight (g)	27.4 ± 0.6	26.9 ± 0.5	> 0.44
Heart rate (beats/min)	433 ± 10	437 ± 7	> 0.26
Right ventricle/ Tibia length (mg/mm)*	1.75 ± 0.06	1.87 ± 0.09	> 0.32
Left ventricle+ septum/ Tibia length (mg/mm)*	5.23 ± 0.14	5.74 ± 0.11	> 0.59

Mean ± SEM analyzed by *t*-test (n=12 or 6*). *P* value vs. WT

Supplementary Figure 1. Genotyping of transgenic mice. Representative example of PCR genotyping in polyacrylamide gel, bp: base pairs (n=12).

Supplementary Figure 2. Distribution of Herpud1. Representative immunohistochemistry images (10X) of paraffin embedded tissue of (**a**) liver, negative control, only with secondary antibody, (**b**) liver, positive control, Herpud1 antibody, (**c**) Herpud1 KO mouse heart, Herpud1 antibody and (**d**) WT mouse heart, Herpud1 antibody. (**e**) Representative immunocyto3hemistry image of NRVM stained with DAPI and antibodies against Herpud1 and α-actinin (400X).

Supplementary Figure 3. Herpud1 siRNA standardization. Representative Western blots for Herpud1 in NRVM incubated with increasing concentrations (0, 100, 150, and 200 nM) of Herpud1 siRNA (a) #1 and (b) #2. Graphs represent the quantification of each bar using β-tubulin as a loading control. Mean ± SEM analyzed using a one-way ANOVA followed by Bonferroni's post-test $*p<0.05$ vs. 0 nM (n=3).

Supplementary Figure 4. Cytosolic Ca^{2+} levels in cultured NRVM treated with proteasome inhibitor MG132. Graph of cytoplasmic Ca^{2+} kinetics and quantification of the area under the curve (n=3), measured in untreated control (UNR) and Herpud1-knockdown NRVM (siHerpud1 #2, 48 h) and in control NRVM preincubated with MG-132 (1 μM, 3h). The black arrow shows the time of histamine addition (100 mM). Mean ± SEM analyzed using one-way ANOVA followed by Bonferroni's post-test. $*p<0.05$ vs. UNR.

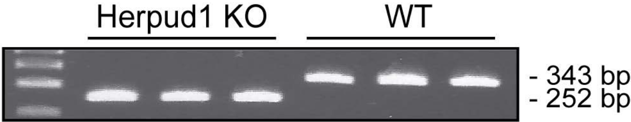
Herpud1 KO
WT
- 343 bp
- 252 bp

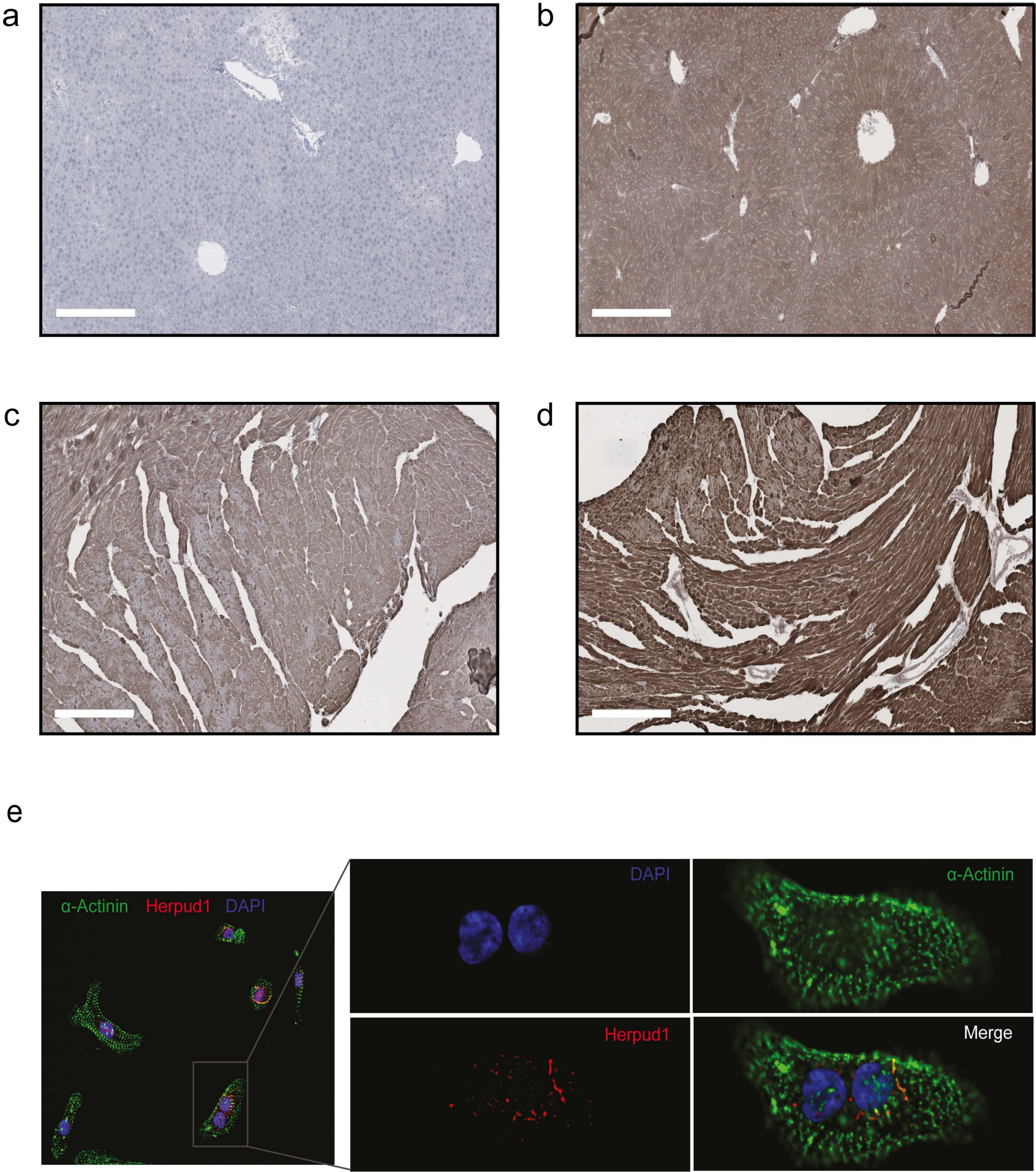
a
b
c
d
e
α-Actinin Herpud1 DAPI
DAPI
α-Actinin
Herpud1
Merge

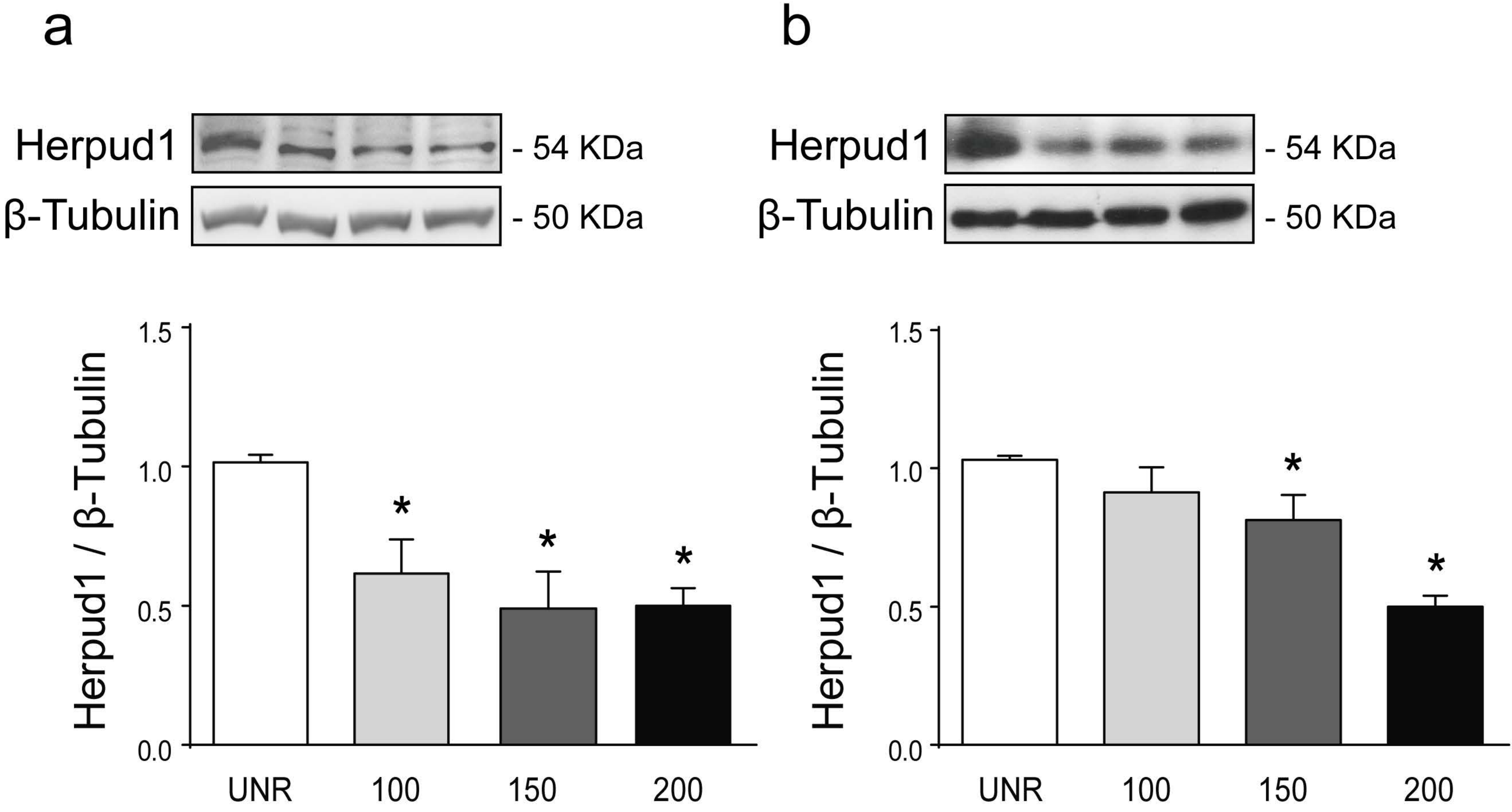
a
b
Herpud1
β-Tubulin
- 54 KDa
- 50 KDa
Herpud1 / β-Tubulin
1.5
1.0
0.5
0.0
*
UNR
100
150
200

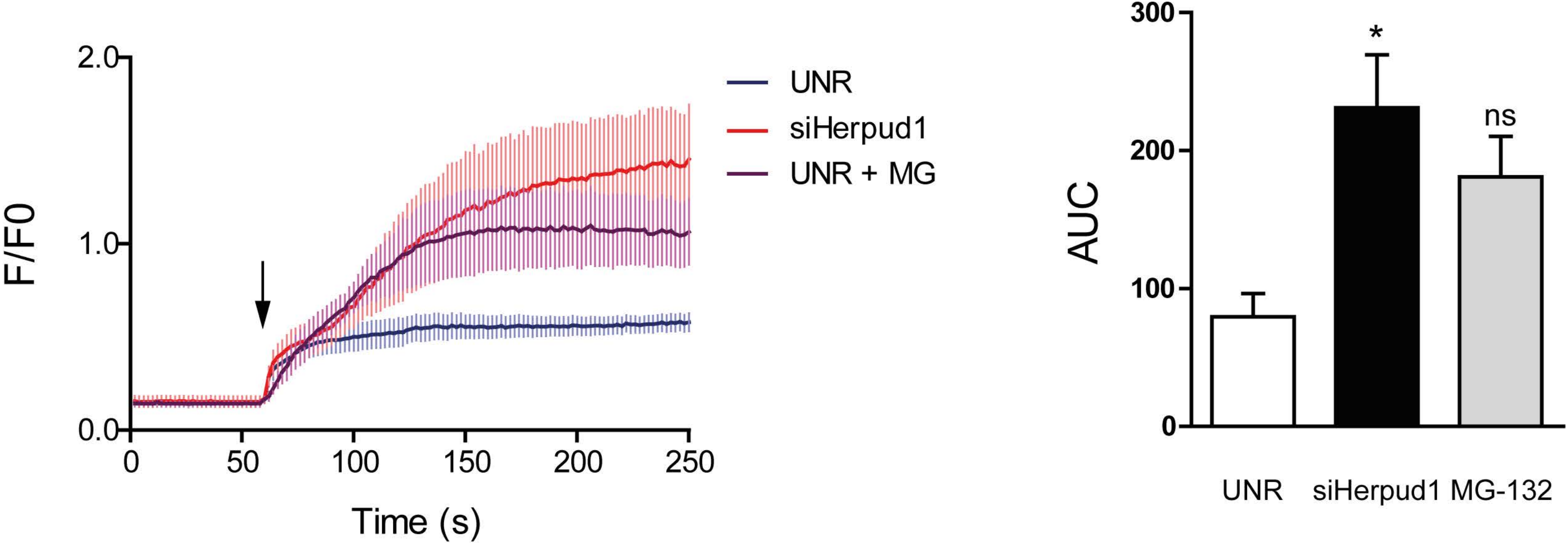

2.0
1.0
0.0
F/F0
0
50
100
150
200
250
Time (s)
UNR
siHerpud1
UNR + MG
300
200
100
0
AUC
*
ns
UNR
siHerpud1
MG-132
